# Comparison of efficacy and accuracy of tooth movements in optimized and conventional attachments of clear aligners - A systematic review and meta-analysis

**DOI:** 10.1016/j.jobcr.2025.07.019

**Published:** 2025-07-29

**Authors:** Srirengalakshmi Muthuswamy Pandian, Aravind Kumar Subramanian, Nikhillesh Vaiid

**Affiliations:** Department of Orthodontics, Saveetha Dental College, Saveetha Institute of Medical and Technical Sciences, India

**Keywords:** Conventional attachment, Optimized attachment, Clear aligner, Orthodontic tooth movement, Accuracy, Efficacy

## Abstract

**Aim:**

This systematic review and meta-analysis aimed to compare the efficacy and accuracy of optimized versus conventional attachments in clear aligner treatment using Invisalign.

**Materials and methods:**

Adhering to the ‘Preferred Reporting Items for Systematic Review and Meta-analysis’ (PRISMA) 2020 guidelines^12^, the review obtained 6 retrospective cohort studies and one randomized controlled trial, sourced from databases such as PubMed, SCOPUS, Web of Science, Cochrane Library, Google Scholar, and clinical trial registries. Four studies were included for meta-analysis. Data was pooled for mean percentage accuracy of various tooth movements and corresponding Forest plots were generated.

**Results:**

Most of the studies included showed a good methodological quality with a low risk of bias. No significant differences in the accuracy of tooth movement between conventional attachments and optimized attachments were noted for canine derotation, anterior extrusion, and root angulation changes in canine, premolar, and molar (p > 0.05). The studies however exhibited high heterogeneity (I^2^ = 75 %). The pooled accuracy for canine derotation was 61.2 % and 71.5 % for conventional and optimized rotations respectively. Similarly, 57.5 % and 62.4 % were the pooled accuracy for conventional and optimized attachments in anterior extrusion. None of the attachments produced the expected tooth movement as predicted by the ClinCheck program.

**Conclusion:**

There was a non-significant difference in accuracy between optimized and conventional attachments for most orthodontic movements. While optimized attachments may offer improved control for specific movements like upper lateral incisor rotation, and conventional attachments potentially enhance anterior extrusion, their overall superiority remains inconclusive. Further high-quality research is needed to validate the hypothesized biomechanical advantages of optimized attachments.

## 1 Introduction

Clear Aligners have traditionally been used to treat minor irregularities in tooth position but in recent years, with the advent of multiple technological tools and rise in digital aids, they have positioned themselves as viable options in treating complex malocclusions.^1^ Clear Aligners provide multiple and varied advantages to patients such as their virtual invisibility, comfort in wearing, and removal during eating and brushing compared to traditional fixed appliances.^2^ Progress in computer-aided design and manufacturing (CAD/CAM) has driven the growing demand for plastic systems in dentistry.^3^ Invisalign is one such thermoplastic clear aligner, introduced and marketed by Align Technology in 1997 and 1999 respectively.^4^ It is the most widely recognized clear aligner system for orthodontists today.^5^

The initial generation Invisalign worked on a ‘displacement-driven system’, which depends on its shape to achieve the desired tooth movement.^6^ No auxiliary elements were incorporated into it. Djeu et al., in 2005, reported a limited efficacy of first-generation Invisalign.^7^ The second-generation Invisalign incorporated auxiliary components, such as composite buttons and inter-maxillary elastics, to enhance orthodontic tooth movement.^6^ The third generation advanced this approach by introducing optimized attachments, automatically placed via the manufacturer's software, which improve tooth movement control by adapting to individual tooth morphology.^6^ They improve the control of tooth movement by adapting their shape to the individual tooth morphology. This Invisalign system with attachments changed the method of use of the aligner over 14 days for 0.25–0.33 mm tooth movement to a weekly aligner swap, reducing the length of treatment by 50 %.^8^

Scientific evidence claims that the predictability is highest for minor horizontal movements such as mesiodistal tipping and labiolingual translation, especially in the upper incisors. In contrast, vertical movements, especially anterior extrusion, and rotation movements of rounded teeth like canines and premolars remain less predictable. While aligners are effective for anterior intrusion and can achieve satisfactory alignment in mild-to-moderate malocclusions, its limitations become apparent in achieving complex bodily movements, torque control, and establishing ideal occlusal contacts. The use of auxiliaries such as attachments, interarch elastics, and optimized aligner geometries is often necessary to improve outcomes.^9^

Earlier, conventional attachments were either rectangular or ellipsoidal, with the latter being the least effective due to its smaller size and limited active surface.^10^ However, conventional rectangular attachments are still widely used. These attachments can be modified in the ClinCheck Pro software based on the clinician's preference in teeth size, prominence, beveling, and position. Smart-Force featured optimized attachments introduced by Align Technology have potentially increased the predictability of tooth movements by improving the accuracy of the biomechanics delivered to the teeth.^11^ ClinCheck program has widely been used to evaluate the accuracy of tooth movement. Though recent systematic reviews have reported the accuracy of various clear aligner attachments, the present review aims to compare the accuracy and efficacy of optimized versus conventional attachments in the Invisalign system.

## 2 Materials and methods

### 2.1 Protocol

The protocol of the present systematic review and meta-analysis was registered in the PROSPERO database with the registered number CRD42024546354. It was designed using the ‘Preferred Reporting Items for Systematic Review and Meta-analysis’ (PRISMA) 2020 guidelines.^12^

### 2.2 Research question

Are optimized attachments more efficient and accurate than conventional attachments of clear aligners for orthodontic tooth movements?

### 2.3 Studies selection criteria

Studies were selected for this systematic review based on the PICO criteria.

### 2.4 PICO criteria

**Population (P):** Adult patients treated with Invisalign aligner system.

**Intervention (I):** Invisalign with optimized attachments.

**Comparison (C):** Invisalign with conventional attachments.

**Outcome (O):** Mean efficacy (predicted – achieved) and mean percentage of accuracy.

### 2.5 Literature search

The databases used to identify studies for the present review include Cochrane Library, Pub Med, Web of Science, SCOPUS, Google Scholar, and trial registries. The search terms and MeSH terms used for search following the PICO principle are presented in Table 1.

**Table 1:** Search strategy.

Search Engine	Search Keywords and MeSH terms
PubMed (49 results)	(“invisible splint" [All Fields] OR “Invisalign" [All Fields] OR “clear aligner" [All Fields] OR “invisible splint attachment" [All Fields] OR “Invisalign attachment" [All Fields] OR “clear aligner attachment" [All Fields]) AND (“optimized" [All Fields] OR “optimized attachment" [All Fields]) AND (“conventional" [All Fields] OR “conventional attachment" [All Fields] OR “horizontal attachment" [All Fields]) AND (“efficiency" [All Fields] OR “efficacy" [All Fields] OR “accuracy" [All Fields])
Cochrane Library (28 results)	#1 (“invisible splint”) OR (“Invisalign”) OR (“clear aligner”) OR (“invisible splint attachment”) OR (“Invisalign attachment”) OR (“clear aligner attachment”) (Word variations have been searched)
	#2 (“optimized”) OR (“optimized attachment”) OR (Word variations have been searched)
	#3 (“conventional”) OR (“conventional attachment”) OR (“horizontal attachment”) (Word variations have been searched)
	#4 (“efficiency”) OR (“efficacy”) OR (“accuracy”) (Word variations have been searched)
	#5 #1 AND #2 AND #3 AND #4
SCOPUS (45 results)	(TITLE-ABS-KEY (“invisible splint” OR “Invisalign” OR “clear aligner” OR “invisible splint attachment”) OR “clear aligner attachment”) OR “Invisalign attachment” AND (TITLE-ABS-KEY (“optimized” OR “optimized attachment”) AND (TITLE-ABS-KEY (“conventional attachment” OR “horizontal attachment”) AND (TITLE-ABS-KEY (“efficiency” OR “efficacy” OR “accuracy”)
Web of Science (28 results)	# 5
	#4 AND #3 AND #2 AND #1
	Indexes = SCI-EXPANDED, CPCI-S, ESCI Timespan = Ten years (2000–2023)
	# 4
	(ALL = (efficiency OR efficacy OR accuracy)) AND LANGUAGE: (All) AND DOCUMENT TYPES: (Article)
	Indexes = SCI-EXPANDED, CPCI-S, ESCI Timespan = Twenty-three years (2000–2023)
	# 3
	(ALL = (conventional OR conventional attachment OR horizontal attachment)) AND LANGUAGE: (All) AND DOCUMENT TYPES: (Article)
	Indexes = SCI-EXPANDED, CPCI-S, ESCI Timespan = Twenty-three years (2000–2023)
	# 2
	ALL = (optimized OR optimized attachment)) AND LANGUAGE: (All) AND DOCUMENT TYPES: (Article)
	# 1
	(ALL = (invisible splint OR Invisalign OR clear aligner OR invisible splint attachment OR Invisalign attachment OR clear aligner attachment)) AND LANGUAGE: (All) AND DOCUMENT TYPES: (Article)

A manual search, assisted by a librarian, was carried out across journals related to orthodontics, dentofacial orthopedics, and dental materials, as well as relevant conference proceedings and trial registries to identify ongoing studies. Specific language filters were not applied, and trials published up to January 2025 were considered. The search included randomized controlled trials, prospective clinical studies, and retrospective studies based on predefined eligibility criteria. Case reports, systematic reviews, and other reviews were excluded.

### 2.6 Literature screening

Duplicate studies were removed using RAYYAN software, an artificial intelligence (AI) driven tool designed specifically for systematic reviews. Two independent primary reviewers (SM and AS) screened titles and abstracts in RAYYAN. Any discrepancies were addressed by a senior reviewer (NV). The full texts of the selected studies were then assessed by primary reviewers (SM and AS), with disagreements resolved by the third reviewer (NV). The agreement between the reviewers screening for the articles was κ = 0.89.

### 2.7 Data extraction

Two reviewers independently (SM and AS) extracted data using a pre-piloted, customized template. The forms were refined based on a pilot test using two studies. Any discrepancies in the data extraction process were resolved through discussion with a third reviewer (NV). There was an agreement between the reviewers with the κ value of 0.88. Missing data were sought from corresponding authors; data were excluded if unavailable after this attempt. The following trial characteristics were noted:

1. Author, publication year, study country, and study design (e.g., retrospective, prospective, clinical trial, randomized controlled trial).
2. Participant's demographic details such as age, gender, and sample size distribution
3. Type of movement performed
4. Intervention (optimized attachments and conventional attachments) type
5. Outcome details and inference

### 2.8 Assessment of risk of bias

The quality of the included studies was assessed as a part of the data extraction process. The revised Cochrane Risk of Bias Tool for Randomized Trials (RoB-2)^13^ was employed to analyze the randomized study. RoB-2 tool assessed the bias arising from the following:

1. Process of randomization
2. Deviations from the intended interventions
3. Missing outcome data
4. Measurement of the outcome
5. Selection of the reported results

Each of the five domains contained two to three subdomains, with signaling questions to be addressed. Responses to these questions determined the following bias risk classifications:

a. Low risk of bias: Studies in which all subdomains were assessed as having “low risk.”
b. Some concerns: Studies in which one or more than one subdomain was assessed as having “some concerns.”
c. High risk of bias: Studies in which one or more subdomains were rated as “high risk” and more than two domains were classified as having “some concerns.“^13^

Joanna Briggs Institute adopted for cohort studies tool was used to assess the quality of retrospective studies.^14^ Criteria such as recruitment, assigning to intervention, valid and reliable measurement tools, identifying confounding factors, strategies to deal with confounders, loss to follow-up, strategies to deal with loss to follow-up, and the statistical test were used. The study was graded as high risk, unclear risk, and low risk based on the total scores.^14^ The agreement between the reviewers in assessing the risk of bias for the included articles was κ = 0.86.

### 2.9 Data synthesis

To compare the effects of conventional and optimized attachments, the mean percentage accuracy was synthesized and expressed as mean differences with standard deviations. A meta-analysis was conducted, incorporating studies reporting similar tooth movements. The pooled effect estimate was calculated as the weighted mean difference using a random-effects model (DerSimonian-Laird method) to generate conservative confidence intervals. The I^2^ statistics was used for heterogeneity assessment.^15^ Funnel plot analysis was used for identification of possible publication related bias.

### 2.10 Quality of evidence assessment

To determine the certainty of evidence The ‘Grading of Recommendations, Assessment, Development, and Evaluation’ (GRADE) approach was applied. The GRADEpro GDT software was utilized, categorizing the evidence quality as very low, low, moderate, or high.^16^

## 3 Results

### 3.1 Study search and selection

**Fig. 1:**
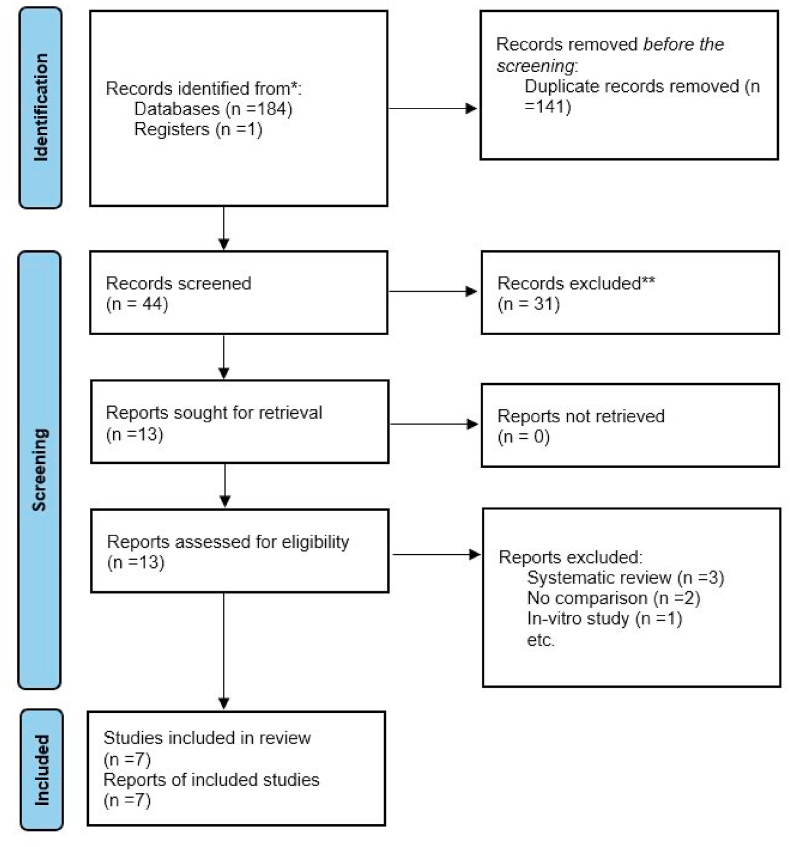
PRISMA 2020 flow diagram.

A comprehensive literature search was conducted across multiple databases and resources, including PubMed, Cochrane Library, SCOPUS, Web of Science, Google Scholar, hand searching, and trial registries, yielding 185 initial records. After the exclusion of 141 duplicates, the abstracts and titles of the remaining 44 studies were subjected to screening. Thirty-one studies were excluded at this stage due to ineligibility. Full-text review was then performed on the 13 remaining studies to determine final inclusion (Fig. 1). Studies on Inter Proximal Reduction,^17^ those with no comparison,^18^ in vitro studies,^19^ and three systematic reviews^20^, ^21^, ^22^ were excluded, with reasons provided in Table 2. Ultimately, 7 studies were included for qualitative synthesis^23^, ^24^, ^25^, ^26^, ^27^, ^28^, ^29^ and 4 for quantitative synthesis^23^, ^24^, ^25^^,^^27^

**Table 2:** List of excluded studies with reason.

Author/Year	Reason for Exclusion
Kravitz et al., 2008	Attachments with interproximal reduction
Costa et al., 2020	No comparison
Ferlias et al., 2022	In-vitro study
Nucera et al., 2022	Systematic review
Alharbi et al., 2023	Systematic review
Jedlinski et al., 2023	Systematic review

### 3.2 Characteristics of the included studies

**T**he characteristics of the seven studies included in this review are summarized as Table 3 Overall 526 patients, aged between 11 and 67 years, were assessed for various orthodontic tooth movements using both optimized and conventional attachments. About 177 males and 349 females were involved. Three studies used conventional and optimized attachments for anterior teeth extrusion,^23^^,^^25^^,^^27^ three studies compared conventional and optimized attachments for rotational movement,^23^^,^^24^^,^^28^ one study for incisor intrusion,^26^ one study for root angulation.^29^

**Table 3:** Characteristics of included studies.

Author/Year	Study Design	Sample size	Age range/Gender	Performed movement	Intervention groups	Results	Inference
Karras et al., 2021; 23	Retrospective cohort study	382 teeth from 100 orthodontic patients	11–63 years Male: 32 Female: 68	Rotational and extrusive tooth movements	Optimized rotation −163 teeth Conventional rotation −72 teeth Optimized extrusion −81 teeth Conventional extrusion −66	**Rotational Movement** Movement Tooth Optimized Conventional |P-A| Canine 4.42 ± 5.61 5.65 ± 5.49 Premolar 4.64 ± 5.73 6.85 ± 7.98 Accuracy (%) Canine 66.9 ± 24.4 59.2 ± 27.4 Premolar 65.7 ± 25.9 53.4 ± 25.9 **Extrusion Movement** Movement Tooth Optimized Conventional |P-A| Incisor 0.48 ± 0.37 0.63 ± 0.39 Canine 0.44 ± 0.51 0.78 ± 0.44 Accuracy (%) Incisor 56.1 ± 26.1 49.9 ± 25.1 Canine 50.5 ± 44.8 25.3 ± 23.4	Conventional attachments may be as effective as optimized attachments for the rotation of canines, premolars, and extrusion of incisors and canines. However, overcorrecting tooth movements is recommended
Stephens et al., 2022; 24	Retrospective cohort study	75 patients	>18 years	Canine rotational	Group 1: Optimized rotation attachments under a 1-week wear schedule (OR1) Group 2: Optimized rotation attachments under a 2-week wear schedule (OR2) Group 3: Conventional beveled rectangular attachments under a 2-week schedule (V2)	Efficiency was calculated with the formula [Achieved change/Predicted change ∗ 100] Median efficiency OR1 = 81.5 % OR2 = 76.5 % V2 = 63.1 %	Vertical rectangular attachments exhibited less predicted rotation compared to optimized attachments
Burashed., 2023 (Open bite); 25	Retrospective cohort study	86 patients	14–57 years Male: 29 Female: 57	Upper/lower incisor extrusion	Group A (42): Conventional attachments Group B (44): Optimized attachments	**Extrusion Movement** Movement Group A Group B |FO-IO| 1.3 ± 1.2 1.6 ± 1.2 Efficacy (%) 54.6 ± 40.3 62.1 ± 33.4	Optimized attachments are more effective in incisor extrusion to correct open bites with shorter treatment times. However, attachment types do not improve the success rate
Burashed et al., 2023 (Over bite); 26	Retrospective cohort study	78 patients	18–67 years Male: 20 Female: 22	Upper/lower Incisor intrusion	Group A (42): Conventional attachments Group B (36): Optimized G5 attachments Both groups are sub-divided into incisor-intrusion alone and incisor intrusion with premolar extrusion	**Overbite Reduction** Movement Group A Group B |FOB-IOB| −1.3 ± 1.2–1.3 ± 1.3 Efficacy (%) 40.3 ± 33.5 36.6 ± 35.4	Optimized attachments are no more effective than conventional attachments in reducing deep overbite. Attachment type does not improve the success rate. Overcorrection is recommended
Groody et al., 2023; 27	Randomized clinical trial	71 maxillary lateral incisors from 38 patients	18–50 years Male: 11 Female: 27	Lateral incisor extrusion	Optimized group (O) (n = 23) Horizontal non-beveled group (H) (n = 20) Horizontal Incisally-beveled group (HIB) (n = 15) Horizontal gingivally-beveled group (HGB) (n = 16)	**Extrusion Movement** Accuracy (%) Optimized Conventional 62 % 76 %	Horizontal attachments are more effective than optimized attachments for extruding maxillary lateral incisor
Hassanaly et al., 2024; 28	Retrospective cohort study	187 upper incisors from 95 patients	Mean age in years: 44.18 ± 4.125 Male: 40 Female: 55	Extrusion/intrusion Relative extrusion/intrusion Vestibular/lingual translation Mesial/distal translation Mesial/distal rotation Mesial/distal angulation Vestibular/lingual inclination	central incisor-optimized attachment central incisor conventional attachment Lateral incisor-optimized attachment Lateral incisor conventional vertical attachment Lateral incisor conventional attachment	Optimized attachment increases the rotation accuracy compared to conventional attachment Conventional vertical attachment increases the mesio-distal angulation accuracy compared to optimized attachment Conventional attachment increases the vestibulo-lingual attachment accuracy compared to optimized attachments	Optimized attachments rotate better lateral incisors; conventional attachment improves mesio-distal angulation and torque movements
Thilagalavanian et al., 2024; 29	Retrospective cohort study	86 premolars from 54 patients	Mean age in years: 27.14 ± 8.46 Male: 15 Female:39	Root angulation in canine, premolar, and first molar teeth adjacent to first and second premolar extraction sites in maxilla	28 first premolar extractions 26 second premolar extractions **Upper first premolar** **Canine** Conventional (n = 32) Optimized (n = 15) **Premolar** Conventional (n = 29) Optimized (n = 18) **Molar** Conventional (n = 37) Optimized (n = 10) **Upper second premolar** **Canine** Conventional (n = 23) Optimized (n = 18) **Premolar** Conventional (n = 22) Optimized (n = 18) **Molar** Conventional (n = 38) Optimized (n = 3)	Variables Teeth (n) AC- PC P value U4 Canine Conventional 32 7.876 ± 8.05 0.235 Optimized 15 5.66 ± 4.48 Premolar Conventional 29 3.96 ± 5.29 0.878 Optimized 18 3.09 ± 3.03 Molar Conventional 37 6.22 ± 7.88 0.064 Optimized 10 1.21 ± 5.00 U5 Canine Conventional 23 5.76 ± 8.59 0.716 Optimized 18 5.01 ± 4.20 Premolar Conventional 22 1.24 ± 3.40 0.292 Optimized 18 2.83 ± 5.89 Molar Conventional 38 7.84 ± 6.17 0.737 Optimized 3 9.09 ± 5.93	Optimized and conventional attachments performed the same in root angulation of teeth adjacent to extraction sites of premolars in the upper arch

### 3.3 Methodological quality assessment of included studies

**Fig. 2:**
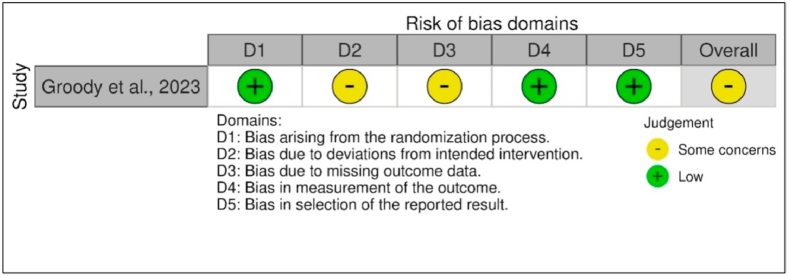
Risk of bias graph (RoB–2).

The quality assessment of the RCTs, conducted using the Revised Cochrane Risk of Bias (RoB-2) tool, is illustrated in the risk of bias graph in Fig. 2. The RoB-2 evaluation of one trial identified concerns regarding deviations from the planned interventions and the potential for bias due to missing outcome data.^27^

The quality assessment for retrospective cohort studies using Joanna Briggs Institute adopted for cohort studies tool is tabulated as Table 4. The 6 involved studies showed low over all risk of bias.^23^, ^24^, ^25^, ^26^^,^^28^^,^^29^

**Table 4:** Risk of bias for retrospective cohort studies.

Study ID	Were the two groups similar and recruited from the same population?	Were the exposures measured similarly to assign people to both exposed and unexposed groups?	Was the exposure measured in a valid and reliable way?	Were confounding factors identified?	Were strategies to deal with confounding factors stated?	Were the groups/participants free of the outcome at the start of the study (or at the moment of exposure)?	Were the outcomes measured in a valid and reliable way?	Was the follow up time reported and sufficient to be long enough for outcomes to occur?	Was follow up complete, and if not, were the reasons to loss to follow up described and explored?	Were strategies to address incomplete follow up utilized?	Was appropriate statistical analysis used?	Overall risk of bias
Karras et al., 2021	Yes	Yes	Yes	Yes	Yes	Yes	Yes	Yes	Yes	Yes	Yes	Low
Stephens et al., 2022	Yes	Yes	Yes	Yes	No	Yes	Yes	Yes	Yes	Yes	Yes	Low
Burashed et al., 2023	Yes	Yes	Yes	Yes	No	Yes	Yes	Yes	Yes	Yes	Yes	Low
Burashed et al., 2023	Yes	Yes	Yes	Yes	No	Yes	Yes	Yes	Yes	Yes	Yes	Low
Hassanaly et al., 2024	Yes	Yes	Yes	Yes	No	Yes	Yes	Yes	Yes	Yes	Yes	Low
Thilagalavanian et al., 2024	Yes	Yes	Yes	Yes	Yes	Yes	Yes	Yes	Yes	Yes	Yes	Low

### 3.4 Meta-analysis

**Fig. 3:**
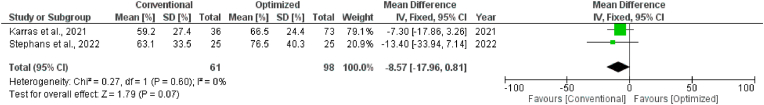
Forest plot showing pooled data of mean percentage accuracy of canine rotation.

**Fig. 4:**
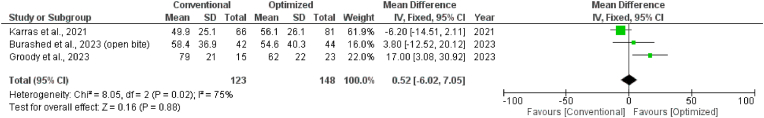
Forest plot showing pooled data of mean percentage accuracy of anterior extrusion.

Two studies that compared conventional and optimized attachments for canine rotation were included for meta-analysis.^23^^,^^24^ Studies that assessed the mean percentage of accuracy using ClinCheck program software and Kravitz formula: 100 − [(|predicted-achieved|)/|predicted|] × 100 were used. Similarly, three studies that assessed the mean percentage accuracy for anterior teeth extrusion were included for the meta-analysis.^23^^,^^25^^,^^27^ The Forest plot for canine de-rotation showed no significant mean difference between conventional and optimized attachment (SMD: −0.31; p-value: 0.06) (Fig. 3) with no heterogeneity (*I*^*2*^ = 0 %). Forest plot for anterior teeth extrusion also showed no significant mean difference in the accuracy (SMD: 0.14; p-value: 0.60) (Fig. 4) with high heterogeneity (*I*^*2*^ = 76 %). Sensitivity analysis of anterior teeth extrusion Forest plot showed no difference in overall effect size and heterogeneity.

### 3.5 Publication bias

**Fig. 5:**
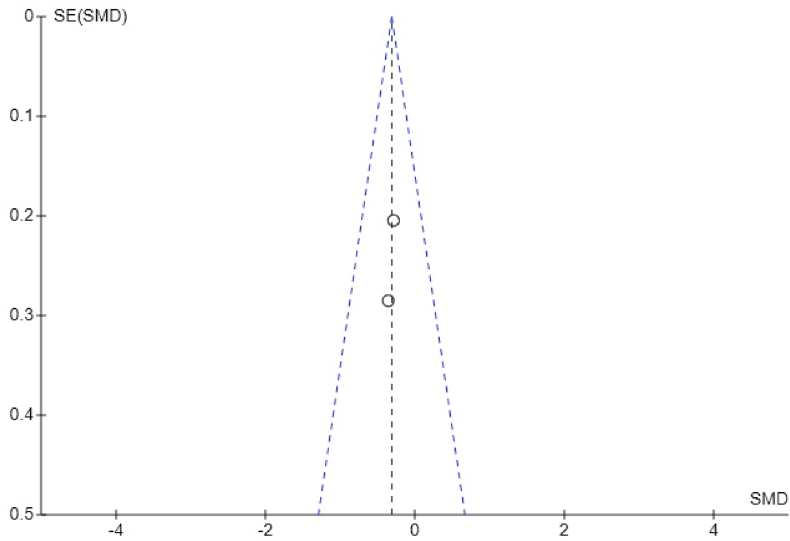
Funnel plot showing publication bias in analysis of mean percentage accuracy of canine rotation.

**Fig. 6:**
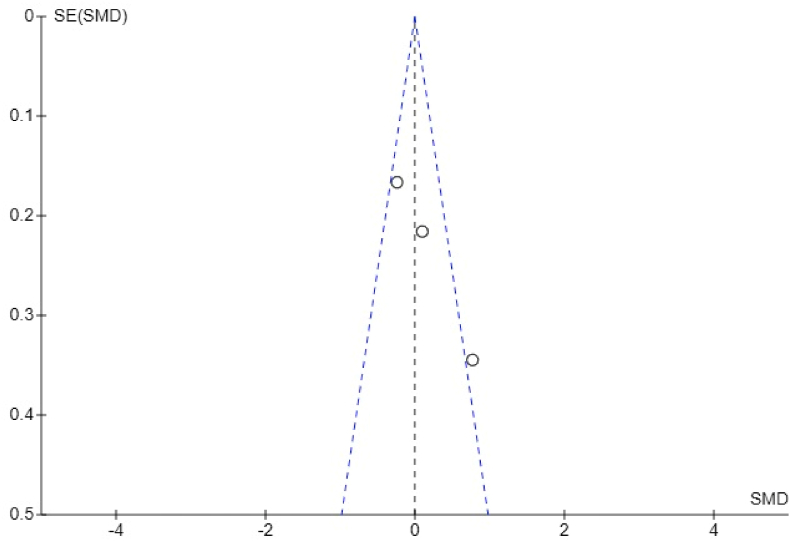
Funnel plot showing publication bias in analysis of mean percentage accuracy of anterior extrusion.

The Funnel plot showing analysis of mean percentage accuracy of canine rotation using optimized and conventional attachments suggests there is no significant publication bias, indicated by a low standard error observed between the sample estimates and true population values (Fig. 5). However, for the anterior teeth extrusion, there is a significant publication bias indicated by a high standard error observed between the sample estimates and true population values in Groody et al.^27^ (Fig. 6).

### 3.6 Certainty of evidence

The assessment for certainty of evidence indicated low certainty for canine de-rotation and high certainty for anterior teeth extrusion (Table 5, Table 6).

**Table 5:** GRADEpro assessment of the certainty of evidence of canine derotation.

Certainty assessment							№ of patients		Effect		Certainty	Importance
№ of studies	Study design	Risk of bias	Inconsistency	Indirectness	Imprecision	Other considerations	Conventional	Optimized	Relative (95 % CI)	Absolute (95 % CI)		
New Analysis												
2	non-randomised studies	not serious	not serious	not serious	not serious	none	61	98	–	SMD **0.31 lower** (0.64 lower to 0.02 higher)	⨁⨁◯◯ Low	CRITICAL

**CI:** confidence interval; **SMD:** standardised mean difference.

**Table 6:** GRADE pro assessment of the certainty of evidence of anterior teeth extrusion.

Certainty assessment							№ of patients		Effect		Certainty	Importance
№ of studies	Study design	Risk of bias	Inconsistency	Indirectness	Imprecision	Other considerations	Conventional	Optimized	Relative (95 % CI)	Absolute (95 % CI)		
New Analysis												
3	randomised trials	not serious	not serious	not serious	not serious	none	123	148	–	SMD **0** (0.24 lower to 0.24 higher)	⨁⨁⨁⨁ High	CRITICAL

**CI:** confidence interval; **SMD:** standardised mean difference.

## 4 Discussion

In 2005, fixed appliances were found to be superior to Invisalign in achieving excellent sagittal discrepancies and occlusal relationships.^30^ While clear aligners demonstrate similar effectiveness to fixed appliances in treating mild to moderate malocclusions, fixed appliances may offer a more favorable approach for treating severe malocclusions.^31^ Several systematic reviews have been published on evaluation of clear aligner efficacy against traditional fixed appliances for complex orthodontic tooth movements.^31^^,^^32^ Several have also focused on the effectiveness of clear aligners with attachments for complex tooth movements.^20^ The present study is the first to compare and evaluate the efficacy and accuracy of optimized attachments versus conventional attachments in clear aligner treatment.

Predominant studies (6) of this systematic review are retrospective cohort studies, where the data was obtained from the database of private orthodontic practices.^23^, ^24^, ^25^, ^26^^,^^28^^,^^29^ One randomized controlled study recruited the study participants from a university hospital and two private practice offices.^27^ The group of movements assessed in this review are canine, and premolar rotational, upper/lower anterior extrusion, upper/lower incisor intrusion, mesio/distal translation, rotation, root angulation, and vestibular/lingual inclination, and translation. All studies used the ClinCheck program to evaluate the efficacy and accuracy. Six included retrospective cohort studies had good methodological quality with low risk of bias.^23^, ^24^, ^25^, ^26^^,^^28^^,^^29^ However, the randomized trial had some concerns about deviations from intended interventions and missing outcomes.^27^ The meta-analysis that compared the mean percentage accuracy of conventional and optimized attachments for canine de rotation and anterior teeth extrusion reported no significant mean difference (p > 0.05). The certainty of evidence reported high levels of evidence for anterior extrusion and low levels for canine de rotation.

### 4.1 Anterior teeth extrusion

Three studies that evaluated the efficacy of attachments for anterior extrusion^23^^,^^25^^,^^27^ found no significant differences between optimized and conventional attachments. About 54.6 %–76 % of efficacy can be expected with Invisalign to incisor extrusion irrespective of attachment type.^23^ The present review also found that 57.5 % and 62.4 % of efficacy for upper and lower anterior extrusion, in conventional and optimized attachments. Some studies suggest that increasing the size of the attachment will increase the efficacy.^33^^,^^34^ Earlier Kravitz et al., in their clinical study, found that extrusion is the least accurate (29.6 %) tooth movement with Invisalign.^35^ Of three studies, one study,^27^ reported increased efficacy of lateral incisor extrusion in conventional attachments (76 %) with increased size and sharper edges compared to optimized attachments (62 %). Neither conventional nor optimized attachments achieved the predicted amount of extrusion in all the studies. However, compared to conventional attachments, optimized anterior extrusion attachments may shorten the time for treatment.

### 4.2 Rotational movement

Of three included studies^23^^,^^24^^,^^28^ that evaluated the efficacy of rotational movement using conventional and optimized attachments of Invisalign, two studies^23^^,^^28^ reported a lack of significant difference for the accuracy of canine and premolar derotation. However, one study,^28^ reported a greater accuracy in optimized attachments for upper lateral incisor derotation. This outcome can be attributed to the use of Align Technology's movement table, which provides a range of movements rather than representing precise movements.^28^ For the canine, the overall accuracy of canine derotation ranged from 55 % to 88.8 % with Invisalign, irrespective of the type of attachment. Similarly, Kravitz et al., 2008 reported 35.8 % canine derotation accuracy.^17^ Also, a previous review reported 29.1 %–49.7 % accuracy for premolar and canine derotation using Invisalign.^36^ This systematic review found 61.2 % and 71.5 % accuracy for conventional and optimized attachments for canine derotation with non significant differences (p > 0.05). The predicted amount of derotation was not achieved with conventional and optimized attachments. However, optimized attachments provided root control using 3D controls for position and amount of bevel.^22^ Additionally, optimized attachments feature a specific surface where the aligner applies pressure, while the non-active surface is relieved to prevent interference.^22^

### 4.3 Anterior teeth intrusion

One study which assessed the efficacy of optimized and conventional attachments for incisor intrusion showed no significant difference.^26^ G5 deep-bite optimized attachments have no advantage over conventional attachments for overbite reduction. Only 33–40 % of accuracy can be elucidated for incisor intrusion using Invisalign.^26^ Few other studies that compared Invisalign and fixed appliance therapy also reinforced the inefficacy of Invisalign for overbite reduction.^37^, ^38^, ^39^ Haouili et al. observed that the intrusion of incisors remained a challenge, even with the G5 features, including bite ramps and pressure areas.^40^

### 4.4 Mesio-distal angulation and vestibulo-lingual inclination

A study by Hassanaly et al. comparing the efficacy of optimized and conventional attachments for mesiodistal angulation and vestibulo-lingual inclination on upper lateral incisor reported, that conventional vertical attachment increases mesiodistal angulation and conventional horizontal attachments increase vestibulo-lingual inclination.^28^ These results can be explained using Align Technology's movement table, which offers a range of possible movements rather than exact movement specifications.

### 4.5 Root angulation

One study, examining the accuracy of Invisalign treatment in maxillary premolar extraction cases, found no significant difference (p > 0.05) between conventional and optimized attachments regarding predicted and actual root angulation changes in adjacent canines, premolars, and molars. Regression analysis, adjusting for wear protocol, age, sex, aligner numbers, and presence of attachments on adjacent canines, premolars, and molars, also revealed no significant discrepancy (p > 0.05) between achieved and predicted initial angulation changes in the upper first and second premolar extraction sites.^29^ The observed lack of influence of the wear protocol on the predictability of root angulation changes may be attributable to the fact that, within a two-week wear protocol, most of the tooth movement occurs during the first week.^41^

### 4.6 Strengths and limitations

This is the first systematic review to compare the efficiency and accuracy of optimized and conventional attachments of clear aligners for orthodontic tooth movements. The present systematic review followed the PRISMA 2020 guidelines for transparent reporting of systematic review and meta-analysis.^12^ Considering the specific search strategy, this review design is reproducible. The limitation is that most of the included studies were retrospective cohort studies which reflects a decreased grade of evidence compared to randomized controlled trials.

### 4.7 Clinical significance and future research implications

This systematic review of orthodontic patients undergoing Invisalign therapy with optimized and conventional attachments showed no significant difference in mean percentage accuracy. However, optimized attachments shorten the treatment time and provide root control compared to conventional attachments. Further parallel arm randomized prospective trials following CONSORT guidelines are needed to determine whether optimized attachments perform better than conventional attachments. Confounding variables of size and shape of conventional attachments, age, gender, wear protocol, number of attachments and race are to be considered while designing the trials.

## 5 Conclusion

1. No significant differences in accuracy between optimized and conventional attachments for most orthodontic movements was observed with both failing to achieve 100 % of predicted movements.
2. Optimized attachments showed better control in upper lateral incisor rotation, while conventional attachments may enhance anterior extrusion due to larger size and sharper edges.
3. Root angulation changes, mesio-distal angulation, and vestibulo-lingual inclination varied slightly between attachment types, but differences were not clinically significant.
4. While optimized attachments are biomechanically designed for better force application, their superiority remains inconclusive, highlighting the need for further high-quality randomized controlled trials.

## Author contributions

S.M., A.S and N.V planned and designed the study, S.M being the P.I performed the experiment with A.S, and drafted the manuscript. Additionally, N.V. conducted the editing and final proofreading of the entire document and reviewed the article and contributed to the interpretation. However, all authors critically revised drafts and approved the final work.

## Data availability statement

All the data associated with the systematic review and meta-analysis is presented in the manuscript.

## Human ethics and consent to participate declarations

Not applicable.

## Clinical trial number

Not applicable.

## Funding

This research received no funding.

## Declaration of competing interest

The authors declare that they do not have any conflict of interest.
